# Inhibition of Osteoclastogenesis and Bone Resorption *in vitro* and *in vivo* by a prenylflavonoid xanthohumol from hops

**DOI:** 10.1038/srep17605

**Published:** 2015-12-01

**Authors:** Jing Li, Li Zeng, Juan Xie, Zhiying Yue, Huayun Deng, Xueyun Ma, Chunbing Zheng, Xiushan Wu, Jian Luo, Mingyao Liu

**Affiliations:** 1 Shanghai Fengxian District Central Hospital and East China Normal University Joint Center for Translational Medicine, Shanghai Key laboratory of Regulatory Biology, Institute of Biomedical Sciences and School of Life Sciences, East China Normal University, Shanghai 200241, China; 2 The Center for Heart Development, Key Lab of MOE for Development Biology and Protein Chemistry, College of Life Sciences, Human Normal University, Changsha, Human 410081, China; 3 Department of Molecular and Cellular Medicine, Institute of Biosciences and Technology, Texas A&M University Health Science Center, Houston, Texas 77030, USA

## Abstract

Excessive RANKL signaling leads to superfluous osteoclast formation and bone resorption, is widespread in the pathologic bone loss and destruction. Therefore, targeting RANKL or its signaling pathway has been a promising and successful strategy for this osteoclast-related diseases. In this study, we examined the effects of xanthohumol (XN), an abundant prenylflavonoid from hops plant, on osteoclastogenesis, osteoclast resorption, and RANKL-induced signaling pathway using both *in vitro* and *in vivo* assay systems. In mouse and human, XN inhibited osteoclast differentiation and osteoclast formation at the early stage. Furthermore, XN inhibited osteoclast actin-ring formation and bone resorption in a dose-dependent manner. In ovariectomized-induced bone loss mouse model and RANKL-injection-induced bone resorption model, we found that administration of XN markedly inhibited bone loss and resorption by suppressing osteoclast activity. At the molecular level, XN disrupted the association of RANK and TRAF6, resulted in the inhibition of NF-κB and Ca^2+^/NFATc1 signaling pathway during osteoclastogenesis. As a results, XN suppressed the expression of osteoclastogenesis-related marker genes, including *CtsK, Nfatc1, Trap, Ctr*. Therefore, our data demonstrated that XN inhibits osteoclastogenesis and bone resorption through RANK/TRAF6 signaling pathways. XN could be a promising drug candidate in the treatment of osteoclast-related diseases such as postmenopausal osteoporosis.

Osteoclast are the only cells for bone-resorbing in mammals^1^. Many pathological bone diseases, including postmenopausal osteoporosis, periodontitis, rheumatoid arthritis, lytic bone metastasis, and Paget’s disease, are characterized by progressive and excessive bone resorption^2^. Therefore, identification of agents to block osteoclast differentiation and resorption are the common and successful strategy for the development of therapeutic drugs for the osteoclast-related diseases^3^. However, the drugs for the diseases are far from ideal. For example, the most widely used anti-osteolytic treatment agent bisphosphonates, which inhibit osteoclast resorption by inducing osteoclast apoptosis, are poorly absorbed and can cause damage to the gastrointestinal tract^4^. Denosumab, a humanized monoclonal antibody to RANKL that inhibits osteoclast differentiation, can cause joint and muscle pain in the arms or legs and increase the rates of infections^5^. Estrogen-replacement therapy, often treated for postmenopausal women, has been proven to increase the risk for uterine and breast cancer, blood clots, and heart attack, and its use has been prohibited for long-term treatment^6^. Therefore, there is considerable scientific and public interest in finding alternative agents and treatments for the osteoclast related diseases.

Osteoclasts are unique, multinucleated giant cells, which originate from hematopoietic cells. Osteoclast differentiation is dependent on two cytokines, a tumor necrosis factor (TNF) family cytokine, receptor activator of nuclear factor-κB (NF-κB) ligand (RANKL), and macrophage-colony stimulating factor (M-CSF)^7^,^8^,^9^. M-CSF can stimulate monocyte proliferation and supports its survival^8^. RANKL, which secreted from mesenchymal stem cell and osteoblasts, stimulates monocyte differentiation into osteoclasts^7^,^9^. The interaction of RANKL with its receptor RANK results in a cascade of intracellular events including nuclear factor kappa-light chain enhancer of activated B cells (NF-κB), mitogen-activated protein kinases (MAPKs), ionic calcium, and calcium/calmodulin-dependent kinase by recruiting the adaptor signal protein TNF receptor associated factor (TRAF6)^10^. As a result, a number of osteoclast-related marker gene, including *tartrateresistant acid phosphatase* (*Trap*), *calcitonin receptor* (*Ctr*), *cathepsin K* (*Ctsk*), and *nuclear factor of activated T cells* (*Nfatc1*), are upregulated.

Hops (*Humulus lupulus* L.) are world-widely used raw material in brewing industry, especially for brewing beer. XN is the most abundant prenylflavonoid from hops plant, with a content of 0.1–1% (dry weight)^11^. This compound has attracted much interest due to its proven pharmacologic safety^12^ and its multiple bioactivities, including anti-cancer^13^, anti-diabetes^14^, anti-inflammatory^11^, anti-bacteria and parasite^11^, and hepatic protection^11^. Therefore, improved brewing technology to produces beer with high XN content has been established in the commercial industry^11^.

Recently, it has been reported that XN can inhibit osteoclast-related genes expression in mouse osteoclast cell line RAW264.7 cells^15^, and stimulate osteoblast differentiation in mouse osteoblast MC3T3-E1 cells^16^. However, the precise molecular mechanism of anti-osteoclastogenesis of XN remains unknown, and the effect of XN on pathological bone loss and bone destruction *in vivo* has not yet been well defined. In the present study, using multiple *in vitro* osteoclast differentiation and bone resorption approaches, we demonstrated that XN suppressed RANKL-induced osteoclast formation and function within non-growth inhibitory concentrations. Moreover, we found that XN has inhibitory effects in two osteoclast-related animal models, the ovariectomy-induced bone loss mouse model and RANKL-injection-induced bone resorption model. Furthermore, XN abrogated the binding between RANK and TRAF6, which leading to the inhibition of NF-κB and Ca^2+^/NFATc1 signaling pathway during osteoclastogenesis. As a result, XN suppressed the expression of osteoclastogenesis-related marker genes. Therefore, our data demonstrate that XN suppresses osteoclastogenesis and osteoporosis *in vitro* and *in vivo* through RANK/TRAF6 signaling pathways.

## Materials and Methods

### Regents and antibodies

Xanthohumol (XN), TRIS, Glycine, NaCl, sodium dodecyl sulfate (SDS), and bovine serum albumin (BSA) was obtained from Sigma (St Louis, MO, USA). RAW264.7 cells were the kind gift from Dr Bryant G Darnay (The University of Texas MD Anderson Cancer Center, TX, USA). Penicillin, streptomycin, a-MEM, and fetal bovine serum (FBS) were obtained from Invitrogen (Calbard, CA, USA). NFATc1 antibody is brought from Santa Cruz Biotechnology. All of the other antibodies were bought from Cell Signaling Technology. Bacteria-derived recombinant mouse RANKL (462-TEC) and M-CSF (416-ML) were from R&D Systems.

### Proliferation assay with SRB method

The proliferation effect of XN was determined by SRB method as previously described^17^. The sulforhodamine B (SRB) method is used for cell proliferation and density determination, based on the measurement of cellular protein content^17^. Briefly, the cells (RAW264.7, BMMs and human monocyte cells) were treated with various concentration of XN. After 4 days, all the cells are fixed by the gentle addition of 50 μl of cold 50% TCA (final concentration, 10% TCA) and incubated for 60 minutes at 4 °C. The supernatant is discarded, and the plates are washed five times with tap water and air dried. Sulforhodamine B (SRB) solution (100 μl) at 0.4% in 1% acetic acid is added to each well, and plates are incubated for 10 minutes at room temperature. Unconjugated SRB is washed by 1% acetic acid and then the conjugated SRB is dissolve in 10 mM Tris. Absorbance was measured with a Spectra MAX microplate reader (Molecular Devices).

### BMMs isolation and *in vitro* osteoclast differentiation assay

For mouse primary cell culture, bone marrow cells isolated from mice were cultured as described previously^18^,^19^. Briefly, Bone marrow cells were isolated from flushing the femurs and tibias of 6- to 8-week-old C57BL/6 mice. To generate BMMs, the cells were cultured in α-MEM with 10% FBS containing 20 ng/ml M-CSF. To generate osteoclasts, the BMMs were seeded into 96-well plates and incubated with M-SCF (20 ng/ml) 2–3 days before stimulation with RANKL (30 ng/ml). After 6 or 4 days, cells were fixed and stained for Tartrate-resistant acid phosphatase (TRAP) activity (Sigma). TRAP positive multinucleated cells with more than 5 nuclei were counted as osteoclasts. For human osteoclastogenesis assay, human peripheral blood mononuclear cells (PBMCs) were isolated from healthy donor by Ficoll gradient centrifugation (provide by Shanghai Blood Center). The culture medium consisting of alpha minimal essential medium (α-MEM) supplemented with 10% foetal bovine serum (FBS). For osteoclastogenesis, 5 × 10^5^ PBMCs were seeded in a 96-well plate with 20 ng/ml human CSF1 (Sino Biological Inc, 11792-H08Y). After 36 hours, cells were stimulated with 50 ng/mL human RANKL (R&D, 6449-TEC) and 20 ng/mL human CSF1 for 8–9 days. Medium was changed every two day. Osteoclasts were fixed and stained using the TRAP staining kit (Sigma, 387A-1KT).

### Actin ring-formation assays

The actin ring-formation assay was performed as described previously^6^,^20^. BMMs differentiated in 6 days with RANKL and various concentration of XN. When the osteoclast formed, the cells were fixed with 4% paraformaldehyde for 10 mins at 4^o^C and then stained with 0.1% phalloidine. The images were obtained by laser scanning confocal microscopy (Leica).

### Bone resorption assay

To observe the bone resorption *in vitro*, BMMs were cultured with M-CSF plus RANKL for 6 days with or without different concentration of XN, then the dentin slices were treated with 1N NH_4_OH for 5 minutes and stained with 0.5% toluidine blue.

### PCR amplification of reverse-transcribed RNA

For the semiquantitative RT-PCR analysis, total cellular RNA was extracted from cells using TRIzol reagent (Invitrogen). PCR primers are followed below: mouse TRAP, 5′-GCTGGAAACCATGATCACCT-3′(forward) and 5′-GAGTTGCCACACAGCATCAC-3′(reverse); mouse Cathepsin K, 5′-CTTCCAATACGTGCAGCAGA-3′ (forward) and 5′-TCTTCAGGGCTTTCTCGTTC-3′(reverse); mouse CTR, 5′-TGCAGACAACTCTTGGTTGG-3′ (forward) and 5′-TCGGTTTCTTCTCCTCTGGA-3′(reverse); mouse NFATc1, 5′-TGGAGAAGCAGAGCACAGAC-3′ (forward) and 5′-GCGGAAA- GGTGGTATCTCAA -3′ (reverse); mouse β-actin 5′-GGCTGTATTCCCCTCCATCG-3′ (forward) and 5′-CCAGTTGGTAACAATGCCATGT -3′ (reverse).

### Intracellular Calcium imaging

To detect whether XN can inhibit calcium signaling in osteoclast, we performed a calcium imaging assay as previously described^20^. Briefly, All of the BMMs were incubated with M-CSF (20 ng/ml) in the intracellular calcium imaging experiment. The M-CSF incubated BMMs were pretreated with or without XN (5 uM) for 24 hours, and then incubated with or without RANKL (30 ng/ml) for another 60 hours. After loading with fura-2-acetomethoxy ester (Beyotime) (2 mM) for an additional 60 minutes in the dark at 37 °C, the cells were imaged at 340- and 380-nm excitation to detect intracellular free calcium (Olympus IX71 and LAMBDA DG-4) and recorded by In Vivo software and analyzed by Image-Pro Analyzer 6.2 software (Media Cybernetics). Therefore, the cells in Blank group only have M-CSF treatment, the cells in RANKL group have M-CSF and RANKL stimulation, while the cells in RANKL + XN group have M-CSF, RANKL, and XN treatment.

### Luciferase reporter gene assay

RAW264.7 cells were then transfected with NF-κB -luciferase plasmid and FUGENE reagent (Roche) in 5% serum media for 48 hours and then seeded in a 24-well, treated with RANKL and XN for another 24 hrs. Luciferase activity measured using a luciferase assay kit and a microplate luminometer.

### Co-Imunoprecipitation assay

The coimunoprecipitation assay were performed as previously described^21^,^22^. Briefly, the RAW264.7 cells were seed in a 6-cm dish and pretreat with indicated concentration of XN for 4 hours and then the cells were incubated with RANKL (30 ng/ml) for 20 minutes. The cells lysate supernatant were added with the RANK or TRAF6 antibody. After incubated with the protein A or G agarose beads (Thermo), the immunoprecipitated protein were subjected to Western blot using TRAF6 or RANK antibody.

### Chromatin immunoprecipitation (CHIP) assay

The coimunoprecipitation assay were carried out as previously described^23^. Briefly, RAW264.7 cells were plated in 10-cm dish and treated with 30 ng/ml RANKL for 48 hours and then cross-linked with 1% formaldehyde for 10 min at room temperature before harvest. After sonicated, the sample was pre-cleared with Protein G Agarose/Salmon Sperm DNA (Millipore) at 4 °C for 1 h with rotation, and 1% of the pre-cleared chromatin was set aside as the input control. Immunoprecipitation was carried out with 5 μg of antibody overnight at 4 °C against NFATc1 or Rabbit IgG (Santa Cruz Biotechnology). Immune complexes were pulled down and subjected to quantitative real-time PCR. Results were normalized to the input control. Primer for amplifing the NFATc1 binding regions of the mouse cathepsin K promoter are 5′-CCCCCAAAGTCAGTCAGATG-3 and 5′-GGTAAGGATTGCGGAAGTCA-3′.

### *In vivo* animal model

For the ovariectomy (OVX) animal study, Female 8-week-old C57/BL6 mice were performed ovariectomy or sham-operation. After four weeks, the OVX mice were random divided into two groups: OVX mice treated with vehicle group and OVX mice treated with XN group (n = 6). The mice were injected intraperitoneally with 10 mg/kg XN or vehicle control for another five weeks every day. Then, all the mice were euthanized with excess amounts of anesthetic. Bone histomorphometry was analyzed on lumbar vertebrae as described previously^6^. For the RANKL-injection bone resorption model, RANKL/vehicle and RANKL/XN were injected into the calvaria of 6-week-old C57BL/6 male mice (n = 6) every day for two weeks. The concentration of RANKL is 1 mg/kg and XN is 10 mg/kg. At 15 days after the first injection, the mice were sacrificed and calvaria were collected. Mouse calvaria were collected for TRAP staining or section for TRAP staining (Sigma). All animal experiment procedures were approved by the Animal Care and Use Committee of the East China Normal University according to the animal standards of care of the National Institutes of Health.

### Statistical analysis

All experimental data are presented as the mean SEM. Statistical significance was determined by the Mann-Whitney U test and Student’s t test. Significance was considered at p < 0.01.

## Results

### XN suppresses human and mouse osteoclast differentiation within non-growth inhibitory concentration

**Figure 1:**
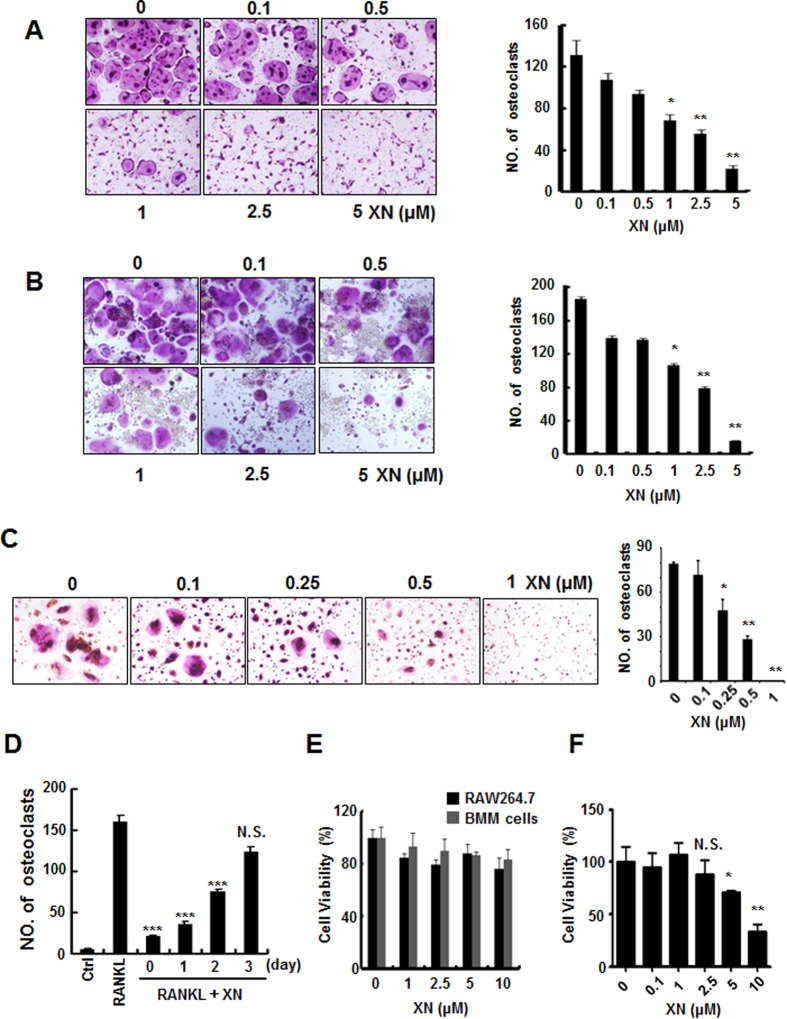
XN suppresses human and mouse osteoclast differentiation. (**A**) The effect of xanthohumol (XN) on mouse BMMs differentiation. BMMs were incubated with M-CSF (20 ng/ml) and RANKL (30 ng/ml), followed by addition of different concentrations of XN for 6 days. The cells were stained for TRAP assay and photographed (×40; left). The numbers of TRAP positive multinucleated (>5 nuclei) osteoclasts were counted (right). (**B**) The effect of XN on RAW264.7 cell differentiation. RAW264.7 cells were treated with RANKL (30 ng/ml) and different doses of XN for 3 days. The cells were stained for TRAP assay and photographed (×40; left) and the numbers of TRAP positive multinucleated (>3 nuclei) osteoclasts were counted (right). (**C**) The effect of XN on human PBMC differentiation. PBMCs (5 × 10^5^ cells) were stimulated with hRANKL (50 ng/mL) and hCSF1 (20 ng/mL) for 8–9 days. The cells were stained for TRAP assay and photographed (×40; left) and the numbers of TRAP positive multinucleated (>3 nuclei) osteoclasts were counted (right). (**D**) XN inhibits RANKL-induced osteoclast differentiation at the early stage. Osteoclast precursor BMMs were cultured with M-CSF and RANKL for differentiation into mature osteoclasts in 6 days. XN (5 μM) were added at indicated time (day). The cells were fixed and stained for TRAP activity. (**E**) The effect of XN on cell viability in BMMs and RAW264.7 cells. BMMs or RAW264.7 cells were treated with different concentrations of XN for 5 days, and the cell viability was measured by SRB assay. (**F**) The effect of XN on cell viability in human PBMC cells. Human PBMC cells were incubated with hCSF1 (20 ng/mL) and different concentrations of XN for 4 days. The cell viability was measured by SRB assay. Column, means of experiments performed in triplicate; bar, SD. *p < 0.05, **p < 0.01, ***p < 0.001. N.S., no significant.

In order to determine the effect of XN on osteoclastogenesis, we employed three standard *in vitro* osteoclast differentiation models, mouse BMMs with RANKL and M-CSF stimulation model, RAW264.7 cells with RANKL stimulation model, and human PBMC cells with RANKL and M-CSF stimulation model. As shown in Fig. 1A–C, TRAP positive multinucleated osteoclasts were formed in response to RANKL treatment. However, administration of XN reduced osteoclast differentiation in a dose-dependent manner in all of the three cell models. The half maximal inhibitory concentration (IC_50_) value of mouse osteoclast is about 1 μM, while the IC_50_ value of human is between 0.25 and 0.5 μM.

To determine at which stage XN blocked osteoclastogenesis, XN was added to osteoclast differentiation cultures beginning on days 0 to day 3 for RAW264.7 cells (Fig. 1D). Our results demonstrate that XN inhibited osteoclastogenesis maximally when added from the beginning with RANKL stimulation (Fig. 1D). The exposure of precursor cells to XN at later stages (more than 2 days) was much less effective in the blockage of osteoclastogenesis. Therefore, our data indicate that XN can suppress RANKL-induced osteoclastogenesis at the early stage and the differentiation process cannot be reversed.

To investigate whether the anti-osteoclastogenesis effect of XN is due to the potential toxicity of this compound, we further examined the cytotoxicity of XN by SRB assay on osteoclast cells (Fig. 1E,F). Our results showed that no significant cytotoxicity of XN was observed at the concentrations used, suggesting that the effects of XN on osteoclastogenesis are not mediated by the toxicity of the compound on cell viability.

### XN blocks RANKL-induced actin-ring formation and bone-resorption activity *in vitro*

**Figure 2:**
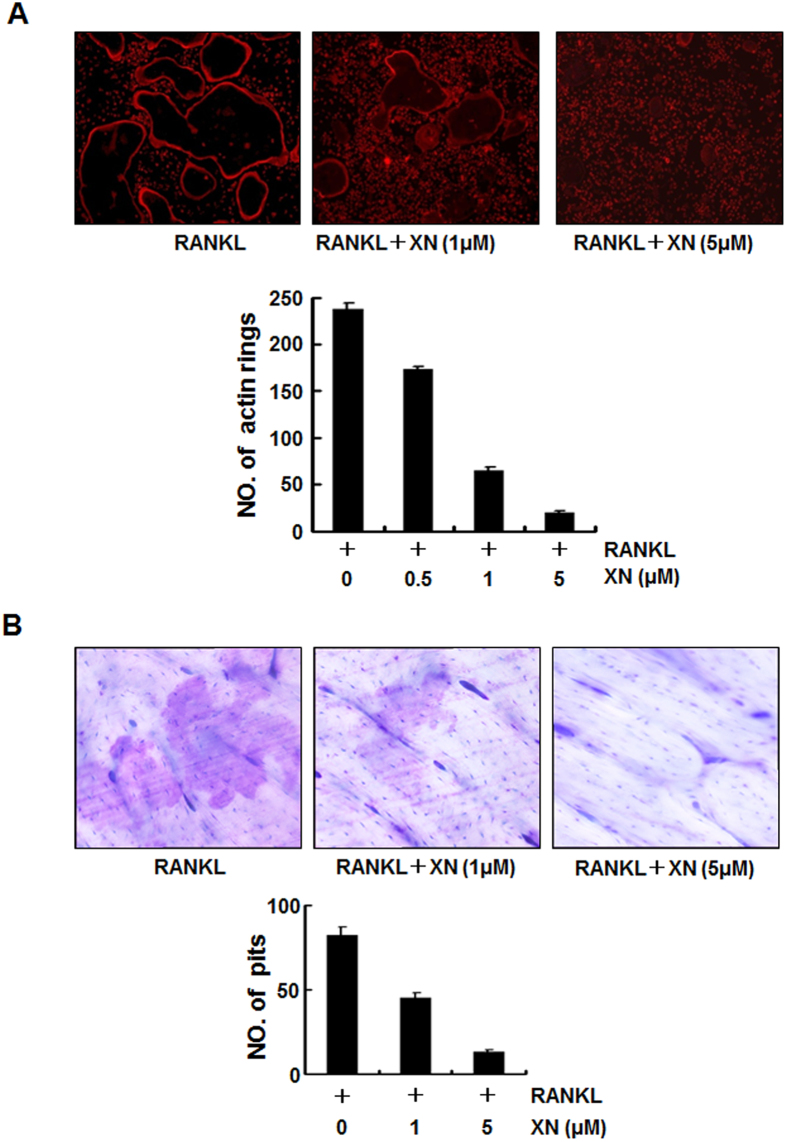
XN inhibits RANKL-induced actin-ring formation and bone resorption. (**A**) The effect of XN on actin-ring formation of osteoclast. Mouse BMMs were incubated with RANKL (30 ng/ml) in the presence of M-CSF (20 ng/ml), followed by treatment with indicated doses of XN. Cells were fixed and stained for F-actin (top). Osteoclasts with actin-rings were counted (bottom). (**B**) The effect of XN on pits formation of osteoclast. Mouse BMMs were cultured with M-CSF (20 ng/ml) and RANKL (30 ng/ml) for 6 days with or without indicated doses of XN. The cells were washed and the resorption pits were stained with Mayer’s hematoxylin and photographed (top, original magnification, ×40). The numbers of pits were analyzed with Image-Pro software (bottom). Column, means of experiments performed in triplicate; bar, SD.

To further examine the effects of XN on osteoclastogenesis, we examined whether XN affected RANKL-induced osteoclast function by bone resorption assays. Firstly, we tested whether XN could affect the actin-ring formation, which is a prerequisite for osteoclast bone resorption and is the most obvious character of mature osteoclast during osteoclastogenesis^6^,^18^. In the presence of RANKL exposure, BMMs can differentiate into mature osteoclasts and form distinct actin-ring structures (Fig. 2A, left). However, XN significantly reduced the size and the number of actin-ring structures in a dose-dependent manner, suggesting that XN suppressed the formation of actin-rings by matured osteoclasts.

To further explore whether XN inhibited bone-resorption ability of osteoclast *in vitro*, we performed the pit formation assays using BMMs cells as previously described^20^. As shown in Fig. 2B, osteoclasts caused bone-resorption and pit formation in the present of RANKL (Fig. 2B, left). Addition of XN significantly reduced the density of pits on the surface of dentin slices in a concentration-dependent manner (Fig. 2B). These results suggested that XN suppressed RANKL-induced bone resorption activity.

### XN inhibits ovariectomy-induced bone loss by inhibiting osteoclast activity

**Figure 3:**
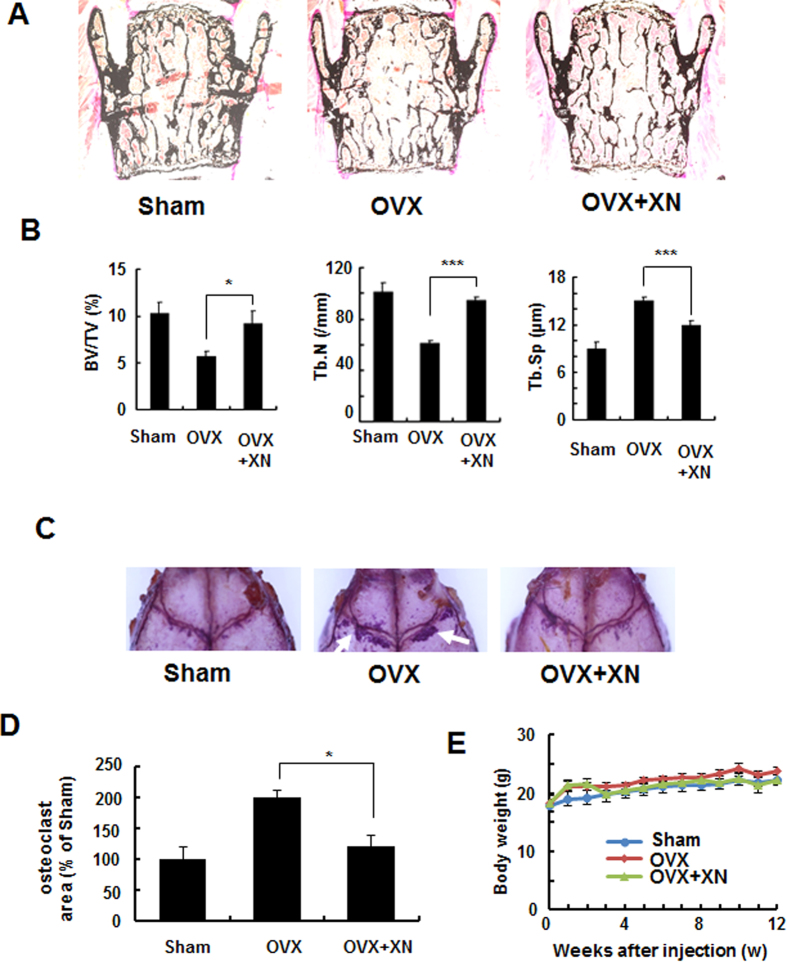
XN inhibits ovariectomy-induced bone loss by inhibiting osteoclast activity *in vivo*. Four weeks after ovariectomy or sham-operation, mice were divided into three groups: sham-operated mice (sham), ovariectomized mice treated with vehicle (OVX) and OVX mice treated with XN (OVX + XN) (10 mg/kg, n = 6) for another five weeks. The treated mice were intraperitoneally (i.p.) injected with XN every days. Then, all the mice were euthanized for bone histomorphometry. (**A,B**) Histomorphometric analysis of lumbar vertebrae from sham, OVX, OVX + XN mice. Bone value/total value (BV/TV), trabecular space (Tb.Sp), and trabecular number (Tb.N) were analyzed as described in Materials and Methods. n = 6. (**C,D**) TRAP staining of the whole calvaria. The osteoclast area were analyzyed by OsteoMeasure Analysis system as described in Materials and Methods (**D**). (**E**) Effect of XN on mouse body weight at the concentrations tested. *p < 0.05, **p < 0.01, ***p < 0.001.

To further test the possible efficacy in the treatment of pathological bone loss, we examined the effect of XN in post-ovariectomy osteoporosis using a therapeutic experimental protocol. Administration of XN started four weeks after ovariectomy (OVX) and treated for five weeks (Fig. 3A). Histomorphometric analysis of lumbar vertebrae showed that the trabecular bone volume (BV/TV) and trabecular number (Tb.N) were significantly increased in XN treated mice compared to vehicle-treated OVX mice, while trabecular separation (Tb.Sp) was decreased relative to vehicle-treated OVX mice (Fig. 3B, right), suggesting that treatment of XN significantly inhibited the ovariectomy-induced bone loss. To explore whether XN suppressed bone loss through the inhibition of osteoclastogenic activity *in vivo*, we performed TRAP staining on the calvaria bone and quantified the TRAP positive area by software. Our data showed that treatment of OVX mice by XN dramatically decreased the OVX-induced osteoclast activity (Fig. 3C,D), indicating that XN could inhibit ovariectomy-induced osteoclast activity *in vivo*. Moreover, XN had little effect on body weight at the concentrations tested (Fig. 3E), suggesting little toxicity of XN at the tested doses *in vivo*.

### XN abrogates RANKL-injection-induced bone resorption of osteoclast *in vivo*

**Figure 4:**
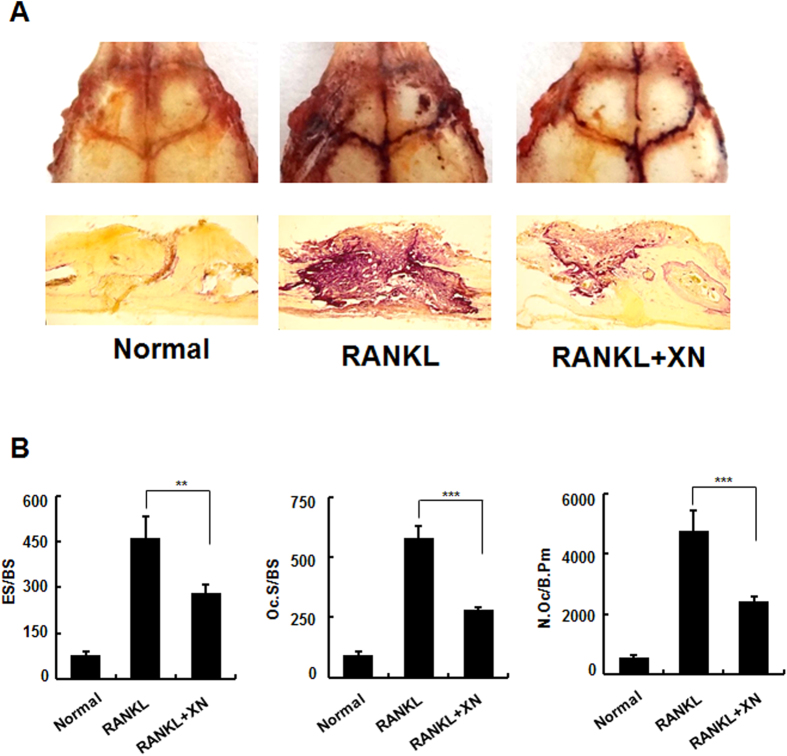
XN prevents RANKL-injection-induced osteoclast activity. RANKL/vehicle and RANKL/XN were injected into the calvaria of 6-week-old male mice every day, respectively (n = 6). After 15 days, the mice were sacrificed and calvaria were stained by TRAP staining and sectioned. (**A**) Representative TRAP stained whole calvaria (top) and calvarial section (bottom) from normal mice, RANKL/vehicle injected mice (RANKL) and RANKL/XN injected mice (RANKL + XN). (**B**) Eroded surface/bone surface (ES/BS); osteoclast surface/bone surface (Oc.S/BS); osteoclast number/bone perimeter (N.Oc/B.Pm) were analyzed by OsteoMeasure Analysis system as described in Materials and Methods.

To further confirm the observation that XN inhibits bone resorption of osteoclast *in vivo*, we employed the extensively used RANKL-injection-induced bone resorption mouse model^2^,^12^. Our results showed that RANKL-injection dramatically induced the activity of osteoclast (Fig. 4A, middle), however administration of XN strikingly suppressed the RANKL-injection-induced osteoclast activity (Fig. 4A, right). Histomorphometric analysis (RANKL + XN versus RANKL mice) indicated that XN inhibited the RANKL-injection-induced bone resorption *in vivo*, including the osteoclast surface/bone surface (Oc.S/BS), eroded surface/bone surface (ES/BS), and osteoclast number/bone perimeter (N.Oc/B.Pm) (Fig. 4B).

### XN inhibits RANKL-induced Ca^2+^/NFATc1 signaling pathway

**Figure 5:**
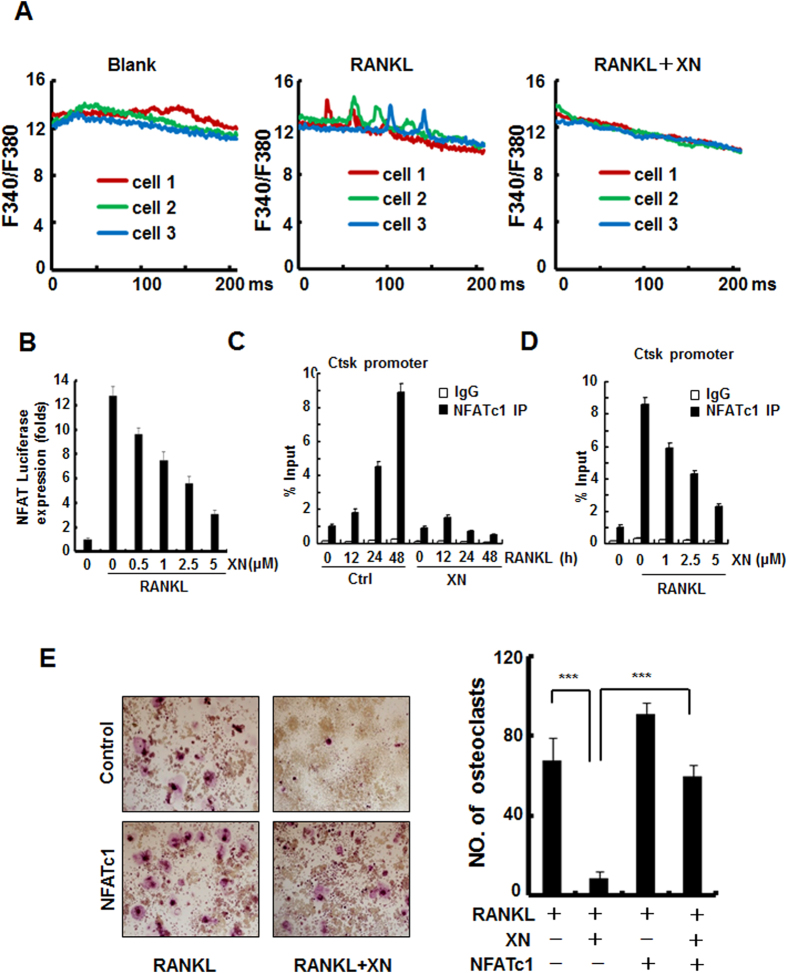
XN blocks RANKL-induced Ca^2+^/NFATc1 signaling pathway. (**A**) The effect of XN on RANKL-induced Ca^2+^ oscillation. The M-CSF incubated BMMs were pretreated with or without XN (5 uM) for 24 hours, and then incubated with or without RANKL (30 ng/ml) for another 60 hours. The Ca^2+^ oscillation was analyzed as described in Materials and Methods. (**B**) The effect of XN on RANKL-induced activity of NFAT. RAW264.7 cells were cotransfected with NFAT-luciferase reporter gene and Renilla gene. After 36 hours, the cells were treated with RANKL and indicated concentrations of XN for another 24 hours. Cell extracts were collected and luciferase activity was measured as described in Materials and Methods. Results are expressed as fold activity over the activity of the control. (**C,D**) The effect of XN on NFATc1 binding to Cathepsin K (Ctsk) promoter region by Chromatin immunoprecipitation (ChIP) assays. BMMs incubated with RANKL (30 ng/ml) were treated with or without XN for indicated time (**C**). Or BMMs incubated with RANKL (30 ng/ml) were treated with indicated concentration of XN for 48 hours (**D**). The Chromatin DNA were immunoprecipitated with control IgG or the anti-NFATc1 antibody and subjected to quantitative real-time PCR with primers specific for NFATc1-binding sites. (**E**) NFATc1 prevents the inhibitory effect of XN in RANKL-induced osteoclast differentiation. RAW264.7 cells were transfected with NFATc1 or vector control plasmids, and then incubated with or without XN (5 μM) in the presence of RANKL (30 ng/ml). After 4 days, the cells were fixed and stained for TRAP activity (left). Original magnification, ×40. The numbers of TRAP positive multinucleated (>3 nuclei) osteoclasts were counted (right). Column, means of three experiments conducted in triplicate; bar, SD. ***P < 0.001.

RANKL-induced Ca^2+^/NFATc1 signaling activation is among the very early molecular events in osteoclastogenesis^6^. Our observations indicated that XN inhibited RANKL-induced osteoclast differentiation at an early stage. Therefore, we employed three separate approaches to investigate whether XN inhibits RANKL-induced Ca^2+^/NFATc1 signaling pathway. First, using calcium imaging assay, we examined whether XN suppressed RANKL-induced Ca^2+^ oscillations. Our results showed that XN at 5 μM completely diminished the amplitude and frequency of Ca^2+^ oscillations induced by RANKL (Fig. 5A). Second, using NFAT-luciferase reporter gene assay, we found that the activity of NFAT, which is the downstream transcription factor of Ca^2+^ oscillation and the master regulator of osteoclastogenesis, was dose-dependently suppressed by XN in response to RANKL stimulation (Fig. 5B). Finally, using ChIP assay, XN concentration- and time-dependently suppressed the recruitment of NFATc1 on Ctsk promoter. To further confirm that XN inhibited osteoclast differentiation by suppressing RANKL-induced NFATc1 activity, we assessed whether overexpression of NFATc1 could rescue the XN-induced suppression of osteoclast differentiation. Our results showed that XN inhibited osteoclast formation in control RAW264.7 cells, but had little inhibitory effect on osteoclastogenesis in NFATc1-overexpressed cells (Fig. 5E), suggesting that NFATc1 prevented XN induced inhibition of osteoclastogenesis. All of the results demonstrate that XN suppresses osteoclast formation by inhibiting Ca^2+^/NFATc1 signaling pathway.

### XN inhibits RANKL-induced NF-κB signaling pathway but not MAPK/AP-1 signaling

**Figure 6:**
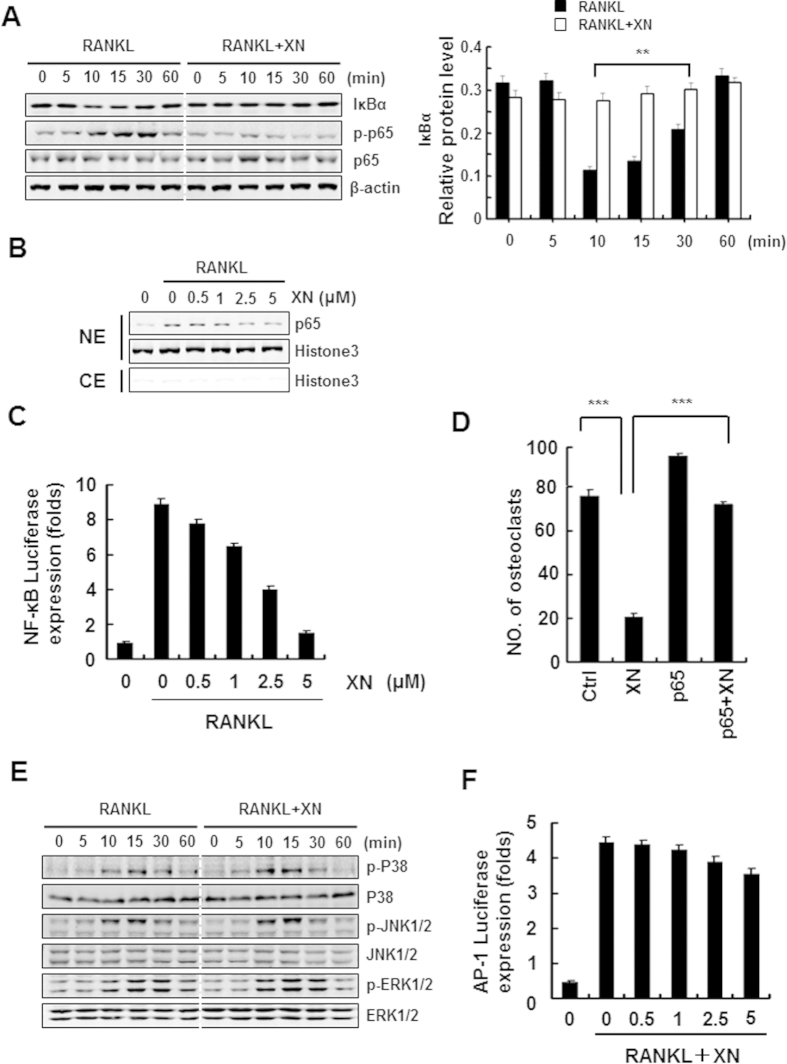
XN suppresses RANKL-induced NF-κB signaling pathway, but has little effect on MAPK/AP-1 signaling. (**A**) The effect of XN on RANKL-induced IκBα degradation and p65 phorsphorylation. RAW264.7 cells were pretreated with XN (5 μM) for 3 hours, and then stimulated with RANKL (30 ng/ml) for indicated time. The degradation of IκBα and the phosphorylation of p65 were tested by Western blot analysis (left). The Western blot were performed in triplicate. The IκBα protein level (with β-actin for normalization) were quantified by Quantity One software (right). (**B**) The effect of XN on RANKL-induced p65 nuclear translocation. RAW264.7 cells were pretreated with different doses of XN for 3 hours, and then stimulated with RANKL for 20 minutes. Cell Nuclear Extracts (NE) and Cytoplasmic Extract (CE) were collected and subjected to Western blot analysis with the indicated antibodies. (**C**) The effect of XN on RANKL-induced activity of NF-κB. RAW264.7 cells were co-transfected with NF-κB-luciferase reporter gene and Renilla gene. After 48 hours, the cells were treated with RANKL and indicated concentrations of XN for another 24 hours. Cell extracts were collected and luciferase activity was measured. (**D**) NF-κB (p65) prevents the inhibitory effect of XN in RANKL-induced osteoclast differentiation. RAW264.7 cells were transfected with p65 or control plasmids, and then incubated with or without XN (5 μM) in the presence of RANKL (30 ng/ml). After 4 days, the cells were stained for TRAP activity and the numbers of osteoclasts were counted. (**E**) XN has little effect on RANKL-induced phosphorylation of MAPKs. BMMs were cultured in the presence of XN (5 μM) for 4 hours, and then RANKL was stimulated at the indicated time points. Cell lysates were extracted for Western blot analysis with indicated antibodies. (**F**) XN has little effect on the activity of AP-1 induced by RANKL. RAW264.7 cells were co-transfected with AP-1-luciferase reporter gene and Renilla gene. After 48 hours, the cells were treated with RANKL and indicated concentrations of XN for another 24 hours. Cell extracts were collected and luciferase activity was measured. Column, means of experiments conducted in triplicate; bar, SD. *p < 0.05, **p < 0.01, ***p < 0.001.

RANKL-induced NF-κB signaling pathway is another essential early molecular event during osteoclastogenesis^24^,^25^. Therefore, we next examined whether XN had any inhibitory effect on RANKL-induced NF-κB signaling pathway. Using Western blot assay, we verified that XN inhibited the degradation of IκBα and the phosphorylation of NF-κB/p65 induced by RANKL (Fig. 6A). Furthermore, XN suppressed RANKL-stimulated nuclear translocation of NF-κB/p65 in a dose-dependent manner (Fig. 6B). Similar results were obtained by the luciferase reporter gene assay that XN concentration-dependently suppressed RANKL-induced NF-κB activity (Fig. 6C). As expected, NF-κB/p65 prevents the inhibitory effect of XN in RANKL-induced osteoclastogenesis (Fig. 6D). Together, all of the data indicate a role of XN in the modulation of NF-κB signaling pathways.

Besides the activation of NFAT and NF-κB signaling pathway, activation of the MAPK/AP-1 pathway plays a pivotal role in osteoclastogenesis^26^,^27^. Interestingly, our data showed that RANKL-induced phosphorylation of p38, JNK1/2, ERK1/2 were not affected with XN treatment (Fig. 6E). Furthermore, XN has little effect on AP-1-lucifearse reporter gene activity within the osteoclastogenesis inhibitory concentration (Fig. 6F), suggesting that XN had little effect on MAPK/AP-1 signaling.

### XN abrogates RANKL-induced association of RANK and TRAF6

**Figure 7:**
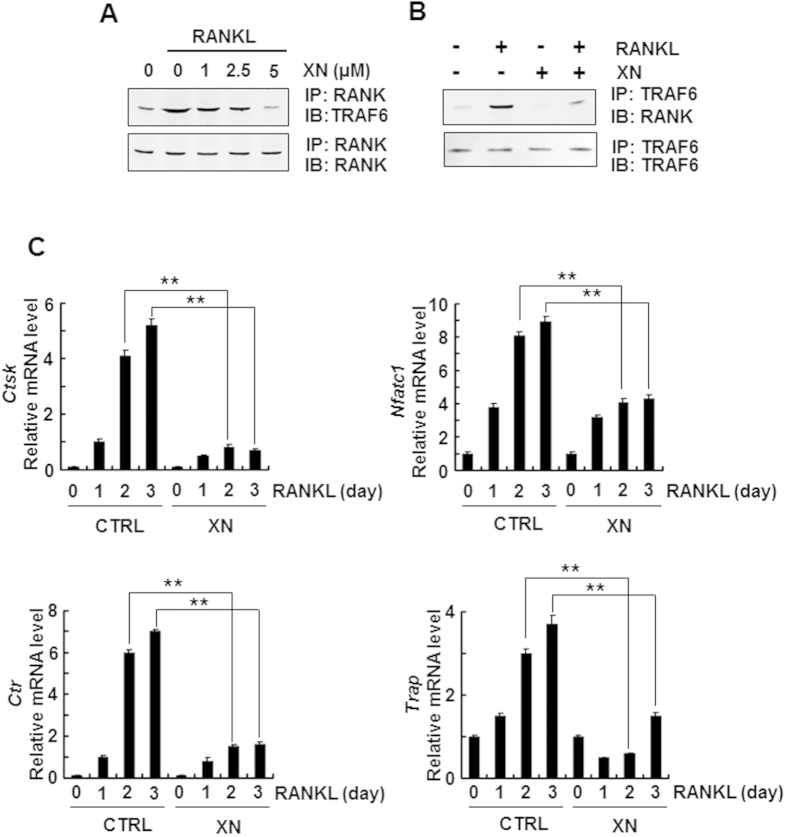
XN represses the association of RANK and TRAF6, and abrogates osteoclastogenesis-related gene expression. (**A,B**) XN suppressed the RANKL-induced binding of RANK and TRAF6. The RAW264.7 cells were pretreated with indicated concentration of XN for 4 hours then stimulated with RANKL (30 ng/ml) for another 20 minutes. The cell lysate were harvest and immunoprecipitated with antibody to RANK and then blotted with anti-TRAF6 (A). Or the cell lysates were immunoprecipitated with antibody to TRAF6 and then blotted with anti-RANK (**B**). (**C**) XN inhibits the mRNA levels of NFATc1, cathepsin K (CtsK), CTR and TRAP induced by RANKL. Mouse BMMs were incubated with XN (5 μM) and RANKL (30 ng/mg) for indicate day. Total RNA was collected and analyzed by Real time-PCR. Column, means of experiments performed in triplicate; bar, SD. **p < 0.01.

Since the NF-κB and Ca^2+^/NFATc1 pathways share the same upstream molecule, TRAF6, which is associated with RANK when activated by RANKL^28^, we then examined the possible effect of XN in the binding between RANK and TRAF6. In the endogenous coimmunoprecipitation assay, XN suppressed RANKL-induced association of RANK and TRAF6 in a dose-dependent manner when immunoprecipitated with antibody to RANK and blotted with anti-TRAF6 (Fig. 7A). On the other end, XN also inhibited the binding when immunoprecipitated with antibody to TRAF6 and blotted with anti-RANK (Fig. 7B). All of the results suggested that XN could dissociate the signal complex RANK and TRAF6.

### XN suppresses RANKL-induced osteoclastogenesis-related marker gene expression

To further confirm the role of XN in osteoclast differentiation, we examined the effects of XN on the osteoclastogenesis-related marker gene expression, including *CtsK, Nfatc1, Trap*, and calcitonin receptor (*Ctr*), all of which are downstream target genes of NF-κB and Ca2+/NFATc1 pathways^29^,^30^,^31^. Our data showed that XN strikingly suppressed the expression of all target genes, including *CtsK, Nfatc1, Trap* and *Ctr* in a time-dependent manner during RANKL-induced osteoclastogenesis (Fig. 7C). Thus, XN is a novel agent to suppress osteoclast differentiation and function.

## Discussion

Hyper-activation of RANKL signaling results in enhanced osteoclast formation and bone resorption, which is common in the pathologic bone destruction, including rheumatoid arthritis, bone tumor and bone metastasis, periodontitis. Therefore, regression of RANL signaling is a proven therapeutic method to the treatment of osteoclast-related diseases^6^. In this report, we demonstrated that XN could inhibit osteoclast differentiation and bone resorption in a dose-dependent manner. In two animal model, XN suppressed bone loss and bone destruction through the suppression of osteoclast activity. Our data also found that XN disrupted the association of RANK and TRAF6, which leaded to the suppression of NF-κB and Ca^2+^/NFATc1 signaling pathway during osteoclastogenesis. As a result, XN blocked the expression of osteoclastogenesis-related marker genes.

Bone is constantly regenerated through continuous formation by osteoblasts and resorption by osteoclasts, which is termed bone remodeling^32^. Previous report demonstrate that XN can stimulate osteoblast differentiation, induce the activity of alkaline phosphatase (ALP), upregulates the osteoblast marker gene Runx2 expression in mouse osteoblast MC3T3-E1 cells^16^. Furthermore, inhibition of p38 and Erk abrogates the XN-induced Runx2 expression during osteoblast differentiation^16^, suggesting XN might stimulate osteoblast differentiation by activating p38 and ERK signaling pathway. In our study, we demonstrated that XN inhibits osteoclastogenesis *in vivo* and *in vitro*. These results indicated that XN could be an attractive therapeutic agent for treating osteoporosis with dual roles in promoting bone formation and inhabiting bone resorption. Interesting, XN has little effect on MAPK signaling pathway in osteoclast, including the phosphorylation of p38, ERK and JNK, suggesting different molecular mechanisms for the effects of XN in bone formation and bone resorption.

It has been reported that RANK/TRAF6 complex is essential for the activation of NF-κB, MAPKs and Ca^2+^ oscillation under RANKL stimulation^33^,^34^. Deficiency of *Traf6* in mice exhibits bone phenotypes similar to *Rankl*^*−/−*^ and *Rank*^*−*/*−*^ in which osteocalstogenesis is largely blocked and the downstream signaling pathways, including NF-κB, MAPKs and Ca^2+^ oscillation, cannot be stimulated by RANKL^33^. However, mutation assays showed that mutation of the membrane-proximal TRAF6 interaction domain in RANK results in completely inhibition of NF-κB signaling, while the MAPK/AP-1 signaling is still possible^33^,^35^,^36^, suggesting that RANK-mediated downstream signaling is domain-dependent^33^. In our study, we found that XN inhibited RANKL-induced activation of NF-κB and Ca^2+^/NFATc1 signaling pathway, but has little effect on MAPK/AP-1 pathway, and XN markedly abrogated the association of RANKL and TRAF6. It is reasonable to speculate that XN targeted the specific TRAF6 binding domain in RANK which is responsible for the activation of NF-κB and Ca^2+^/NFATc1 signaling pathway, but not the MAPK/AP-1 pathway.

Previous studies have demonstrated that NFATc1 is a master regulator during osteoclastogenesis^34^. Activated NF-κB can be recruited to the promoter of NFATc1 and regulates its expression^34^. Calcium signaling leads to nuclear translocate and activate NFATc1 by the Ca^2+^/calmodulin-dependent phosphatase calcineurin^34^. Specially, NF-κB and Ca^2+^oscillation are known to initially induce NFATc1 expression and activity at early stage during osteoclastogenesis. Our results indicate that XN suppresses RANKL-induced osteoclastogenesis only at the early stage by inhibiting the binding of RANK and TRAF6, abrogating NF-κB and Ca^2+^ oscillation signaling, and blocking NFATc1 expression and activity. Therefore, our data strongly suggest that XN inhibit osteoclastogenesis only at the early stage by modulating the RANK/TRAF6 signaling.

Using ovariectomized mouse model and RANKL-injection-induced bone resorption model, our data demonstrate that XN is a promising anti-osteoclastogenesis agent with little toxicity *in vivo*. Many pathological osteoclast-related bone diseases, including periodontitis, rheumatoid arthritis, lytic bone metastasis, and Paget’s disease, are characterized by progressive and excessive osteoclastogenesis and bone resorption. It has been reported that XN also has effect on tumor and inflammation^11^. Therefore, further investigation will be necessary and interesting to examine the effect of XN on periodontitis, rheumatoid arthritis, lytic bone metastasis, and Paget’s disease.

In summary, our data demonstrate that XN can inhibit osteoclastogenesis *in vitro* and *in vivo* through suppressing RANK/TRAF6 interaction, and blocking NF-κB and Ca^2+^/NFATc1 signaling pathway. All of the results suggest that XN is a potential therapeutic agent in the treatment of osteoclast-related diseases such as osteoporosis.

## Additional Information

**How to cite this article**: Li, J. *et al.* Inhibition of Osteoclastogenesis and Bone Resorption *in vitro* and *in vivo* by a prenylflavonoid Xanthohumol from hops. *Sci. Rep.* **5**, 17605; doi: 10.1038/srep17605 (2015).
